# Genetic diversity and population structure of *Vriesea reitzii* (Bromeliaceae), a species from the Southern Brazilian Highlands

**DOI:** 10.1590/1678-4685-GMB-2017-0062

**Published:** 2018-03-19

**Authors:** Luis Eduardo Soares, Márcia Goetze, Camila M. Zanella, Fernanda Bered

**Affiliations:** 1 Universidade Federal do Rio Grande do Sul, Instituto de Biociências, Programa de Pós-graduação em Genética e Biologia Molecular, Porto Alegre, RS, Brazil; 2 Department of Molecular Developmental Biology, Faculty of Science, Radboud Institute for Molecular Life Sciences, Radboud University, Nijmegen, The Netherlands; 3 The John Bingham Laboratory, National Institute of Agricultural Botany (NIAB), Cambridge, UK

**Keywords:** Mixed Ombrophilous Forest, bromeliad, *Araucaria* forest, gene flow, conservation

## Abstract

The Southern Brazilian Highlands are composed by a mosaic of Mixed Ombrophilous Forest (MOF) and grassland formations, an interesting landscape for the study of population structure. We analyzed the genetic diversity within and among populations of the MOF-endemic bromeliad *Vriesea reitzii* by genotyping seven nuclear microsatellite *loci* in 187 individuals from six populations. We characterized levels of genetic diversity and assessed the genetic structure among populations. *Vriesea reitzii* populations showed high levels of genetic variation (number of alleles 28 - 43, allelic richness 3.589 - 5.531) and moderate levels of genetic differentiation (*F*_ST_ = 0.123, *R*_ST_ = 0.096). The high levels of genetic diversity may be explained by species life-history traits, such as habit and mating system. The moderate structure may be a product of the combination of ancient and contemporary gene flow, resulting from the expansion of the forest in the Holocene, and/or due to facilitated dispersal mediated by the MOF’s mosaic landscape. The genetic results indicated no imminent threat to this bromeliad. However, the species is highly associated with the MOF, putting landscape conservation at the center of conservation efforts for the species’ maintenance.

## Introduction

The Brazilian Atlantic rainforest (BAF) represents the second largest tropical moist forest in the world and is recognized for its high levels of biodiversity and endemism (Ledru *et al.*, 2016). The BAF is an ecogeographically subdivided biome. By its broadest definition, it includes several types of vegetation, including semi-deciduous and mixed forests, in addition to the ombrophilous forest of the coast (Oliveira Filho and Fontes, 2000; Goetze *et al.*, 2015). At the southernmost limit of the BAF *sensu lato* lies the Mixed Ombrophilous Forest (MOF), or *Araucaria* Forest, which extends up to 700 km inland (Oliveira Filho and Fontes, 2000). It is disjunctly distributed across the southern plateau of Southeastern Brazil, between 24° and 30° S, at altitudes between 500 and 1400 m above sea level (Behling and Pillar, 2007).

The *Araucaria* Plateau is a geomorphological unit that occupies approximately three-quarters of the southern area of South Brazil, between the Iguaçú and Uruguay Rivers (Behling and Pillar, 2007; Calegari *et al.*, 2017). Its vegetation is characterized by a mosaic of MOF and grassland formations. In the grassland areas it is common to find *Araucaria angustifolia* in an irregular distribution, together with “*capões”* (clumps of trees) and gallery forest, whose floristic composition is similar to that of the MOF (Calegari *et al.*, 2017). The origin of this mosaic, which is characteristic of the Southern Brazilian highlands, has been widely debated. According to Behling *et al.* (2005), several palaeoecological studies carried out in this region have proven that extensive areas of grassland vegetation existed on the highlands throughout the glacial, early and mid-Holocene periods, with forests restricted to deep valley refuges. Mounting evidence suggests that around 3000 years ago, during the Holocene, the MOF expanded from the gallery forests along the rivers as a result of increasing temperatures and humidity (Behling *et al.*, 2004).

Starting around the First World War, fueled by difficulties in commercializing pine wood from Latvia, Europeans began to explore the Brazilian pine *Araucaria angustifolia*. As a consequence, the MOF has suffered a further drastic reduction in its geographic distribution, giving way to the current patchwork of pasture, monoculture stands of *Pinus* and *Eucalyptus*, and open fields interspersed with *Araucaria* fragments (Fonseca *et al.*, 2009; Boelter *et al.*, 2011). In spite of these known – ancient and more recent – factors, the origin of the formation of this mosaic remains unknown, and the effect of human impact on the overall biodiversity of the region throughout time is ill understood.

In fragmented or mosaic landscapes, genetic exchange among plants tends to be restricted, and high genetic differentiation among spatially isolated populations might be expected as a result of random genetic drift, restricted gene flow or selection (Frei *et al.*, 2012; Devitt *et al.*, 2013; Maurice *et al.*, 2016). Studies addressing plant population structure in the MOF are restricted almost exclusively to *Araucaria angustifolia* (e.g., Bittencourt and Sebbenn, 2007; Danner *et al.*, 2013; Medina-Macedo *et al.*, 2015). In order to develop a better understanding of the dynamics of this particular mosaic of forest and grassland in the Southern Brazilian Highlands, the contribution of data from other species is crucial.

The family Bromeliaceae is widespread across the Neotropical region and occupies most distinct environments in the American continent (Brown and Gilmartin, 1989; Smith and Till, 1999; Benzing, 2000; Givnish *et al.*, 2014). Within the BAF, the bromeliads are one of the species richest and most diverse families, therefore representing an important component of this biome (Martinelli *et al.*, 2008). *Vriesea* is the third largest bromeliad genus. It comprises approximately 290 species, 94.8% of which are endemic to Brazil (Costa *et al.*, 2014; Barfuss *et al.*, 2016). The main center of diversity of the genus lies in the BAF (Costa *et al.*, 2014), and most population genetic studies on *Vriesea* focus on species from the coastal regions, or BAF *sensu stricto* (Alves *et al.*, 2004; Palma-Silva *et al.*, 2009; Zanella *et al.*, 2012, 2016). Additional studies have investigated the genetic patterns of bromeliads restricted to higher altitudes of Andean landscape, such as *Puya raimondii* (Sgorbati *et al.*, 2004), as well as tropical inselbergs and outcrops (Barbará *et al.*, 2009; Boisselier-Dubayle *et al.*, 2010; Domingues *et al.*, 2011; Ribeiro *et al.*, 2013; Lavor *et al.*, 2014). However, little is known about species endemic to the MOF.

Here, we investigate the genetic diversity within and among populations of the MOF-endemic bromeliad *Vriesea reitzii* Leme & A.F. Costa, across its entire geographic distribution. *Vriesea reitzii* is an epiphytic bromeliad that occurs in MOF fragments at altitudes ranging from 750 to 1200 m (Rech-Filho *et al.*, 2005; Alves *et al.*, 2006). Due to its morphological similarity with *V. philippocoburgii*, *V. reitzii* was, for a long time, neglected as a species. However, morphological and ecological characteristics have led to the recognition of two distinct taxonomic units (Leme and Costa, 1991). Due to this very recent description of *V. reitzii* as a separate species there is an almost complete lack of data on its breeding system, life history and demographics. However, based on the great resemblance between *V. reitzii* and *V. phillipocoburgii*, it is reasonable to hypothesize that the two species share the same or very similar characteristics. *Vriesea phillipocoburgii* is an epiphyte bromeliad pollinated by hummingbirds, whose seeds are dispersed by the wind (Machado and Semir, 2006; Fischer EA, 1994, Master’s thesis, Universidade Estadual de Campinas). In the only previously published report on the demography and life history of *V. reitzii*, Favretto and Geuster (2012) found that the species bloomed during the spring in the municipality of Joaçaba, Santa Catarina. According to the authors, the species reaches high population densities in places with a good exposure to light.

In addition to the potential conservation implications of our work, this study is motivated by a desire to understand the genetic structure of populations from an extremely fragmented mosaic landscape, the Southern Brazilian Highlands, using *V. reitzii* as model. We hypothesized that the populations of *V. reitzii* would show a high level of genetic structure, similar to other bromeliad species that have been studied on inselbergs. However, there are no reports of genetic population studies in MOF bromeliads, and their genetic structure remains unknown. The specific aims of the present study were: (i) to assess the intra- and inter-population genetic diversity of *V. reitzii* using nuclear DNA; (ii) to quantify the degree of genetic differentiation among populations and discuss the results in the light of the particularity of the forest and grassland mosaic covering the Southern Brazilian Highlands; (iii) to provide data as a basis for conservation actions for the species.

## Material and Methods

### Sampling and DNA extraction

**Figure 1:**
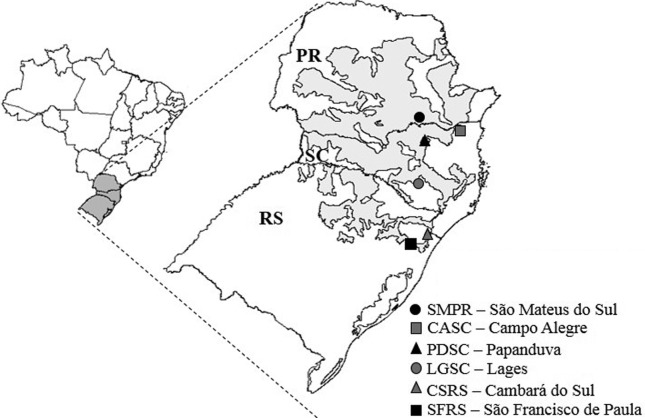
Brazilian map highlighting the southern region with Mixed Ombrophilous Forest in gray. The six *Vriesea reitzii* populations sampled for this study are marked according to legend.

We sampled six populations of *V. reitzii*, covering its entire geographical distribution across the Mixed Ombrophilous Forest. In preparation of the sample collection, we consulted herbarium records and contacted national parks and other locations, for which the species was described. In many of these places, the species was not found anymore, demonstrating the influence of human action on the distribution of *V. reitzii*. We believe that we sampled the main localities where *V. reitzii* currently occurs*.* The minimum distance between populations was approximately 38 km (CFRS to SFRS), with a maximum of about 360 km (CSRS to SMPR). Altitudes ranged from 778 m to 1031 m above sea level (Table 1 and Figure 1). Fresh leaves from 187 flowering or fruiting individuals (approximately 30 per population) were collected and fast dried in silica gel. Total genomic DNA was extracted using the cetyltrimethylammonium bromide (CTAB) protocol, as described by Doyle and Doyle (1990). DNA quantification was performed on a 1% agarose gel stained with GelRed (Biotium, Hayward, CA, USA), in comparison with λ phage DNA.

**Table 1:** Populations of *Vriesea reitzii* sampled, with their vouchers and geographical parameters.

Population Code	Sampling site	Voucher	State*	Coordinates	Altitude (m)
SMPR	São Mateus do Sul	UPCB36191	PR	S25°52’ W50°18’	778
CASC	Campo Alegre	RB00287263	SC	S26°10’ W49°13’	977
PDSC	Papanduva	- §	SC	S26°30’ W50°14’	790
LGSC	Lages	LUSC5573	SC	S27°47’ W50°21’	1031
CSRS	Cambará do Sul	HAS30158	RS	S29°08’ W50°05’	977
SFRS	São Francisco de Paula	HAS66297	RS	S29°26’ W50°36’	927

* Brazilian Federal States: RS, Rio Grande do Sul; SC, Santa Catarina; PR, Paraná.

§ Unable to retrieve voucher from this population. UPBC, Herbarium Departamento de Botânica, Universidade Federal do Paraná; RB, Herbário do Jardim Botânico do Rio de Janeiro; LUSC, Herbarium Lages da Universidade Federal de Santa Catarina; HAS, Herbário Prof. Dr. Alarich Rudolf Holger Schultz, Museu de Ciências Naturais da Fundação Zoobotânica do Rio Grande do Sul.

### Molecular markers and genotyping assays

All samples were genotyped for seven nuclear microsatellite loci described for different bromeliads species: e6B and e19, from *Tillandsia fasciculata* (Boneh *et al.*, 2003); PaA10, from *Pitcairnia albiflos* (Paggi *et al.*, 2008), and VgB10, VgC01 and VgF02, from *Vriesea gigantea* (Palma-Silva *et al.*, 2007). We also genotyped VgF05, an unpublished locus isolated from *V. gigantea* (primers F: TGGGATCATTTCCTTGTTCC, R: CATTCTTGTTTCGCCCAAAT). Amplification reactions were carried out in a TC-412 Thermal Cycler (Techne, Burlington, New Jersey, USA), as described by Palma-Silva *et al.* (2007). The microsatellite alleles were resolved on an ABI 3100 DNA Analyzer Sequencer (Applied Biosystems, Foster City, CA, USA) and sized against the GS500 LIZ molecular size standard (Applied Biosystems) using GENEMARKER Demo version 1.97 (SoftGenetics, State College, PA, USA).

### Data Analyses

The genetic diversity of each population was characterized using the number of alleles (A), number of private alleles (A_P_), allelic richness (*R*_*S*_), expected (*H*_E_) and observed (*H*_O_) heterozygosities, and the inbreeding coefficient (*F*_IS_; Weir and Cockerham, 1984), using the programs FSTAT 2.9.3.2 (Goudet, 1995) and MSA 4.05 (Dieringer and Schlötterer, 2003). To examine departures from the Hardy–Weinberg equilibrium (HWE), exact tests were carried out in GENEPOP 4.0 (Raymond and Rousset, 1995). The data were also tested for genotyping errors resulting from stuttering, short allele dominance, and null alleles using a Monte Carlo simulation of expected allele-size differences implemented in MICRO-CHECKER 2.2.3 (van Oosterhout *et al.*, 2004).

Each population was tested for recent population size reductions (e.g., genetic bottlenecks), using a heterozygosity excess test implemented in the software BOTTLENECK 1.2.02 (Piry *et al.*, 1999). The analysis was carried out using a two-phased mutation model (TPM), with 12% variance and 95% stepwise mutations. Statistical significance was assessed in 10,000 replicates using a one-tailed Wilcoxon signed-rank test. In addition, the occurrence of a genetic bottleneck was tested by estimating the *M*-ratio, the mean ratio of the number of alleles (k) to the range in allele size (r) using the software ARLEQUIN 3.5.2.2 (Excoffier and Lischer, 2010). This method takes advantage of the size specificity of microsatellite allelic states. Significance for each population was assessed by comparison of *M*-ratios with the critical values (*M*_c_ values) obtained by simulating the distribution of *M*-ratios under specific demographic and mutational conditions using the software CRITICAL_M.EXE (http://swfsc.noaa.gov/ textblock.aspx?Division=FED&id=3298) according to Goetze *et al.* (2016). Population genetic differentiation was assessed based on *F*_ST_ (Weir and Cockerham, 1984), on the standardized genetic differentiation measure *G’*_ST_ (Hedrick, 2005), and on Slatkin’s *R*_ST_ (Slatkin, 1995), which estimates the contribution of stepwise-like mutations to genetic differentiation. All these parameters were calculated in the FSTAT software. Pairwise comparisons of *F*_ST_ between populations were carried out using the program ARLEQUIN. The partitioning of genetic diversity within and among populations was examined by Analysis of Molecular Variance (AMOVA; Excoffier *et al.*, 1992) implemented in the software ARLEQUIN. The hypothesis that populations are differentiated because of isolation-by-distance (Wright, 1965) was tested by calculating the correlation between geographic and genetic distance matrices (*F*_ST_), with a standardized Mantel test (Sokal and Rohlf, 1995) using GENEPOP. We also used a Bayesian assignment approach to investigate the population structure of *V. reitzii*, using STRUCTURE 2.3.4 (Pritchard *et al.*, 2000), aiming to assign individuals to genetic clusters (*K*) and to estimate admixture proportions (*Q*) for each individual. The proportion of membership for each cluster was calculated without the consideration of sampling localities. The analyses were carried out under the admixture model assuming independent allele frequencies and using a burn-in period of 250,000, run lengths of 10^6^ and 10 iterations per *K,* for *K* ranging from 1 to 8, to confirm stabilization of the summary statistics (Pritchard *et al.*, 2000). To determine the most likely number of clusters (*K*), we used the method proposed by Evanno *et al.* (2005), which is based on an *ad hoc* measure of Δ*K* that evaluates the second-order rate of change of the likelihood function with respect to *K*. The calculation of Δ*K* was done with STRUCTURE HARVESTER version 0.6.94 (Earl and von Holdt, 2012).

The effective number of migrants (*N*_e_ *m*) between pairs of populations was estimated using a coalescent theory and maximum-likelihood-based approach using MIGRATE 3.0.3 (Beerli and Felsenstein, 1999), as described by Barbará *et al.* (2007). Computations were carried out under both the infinite allele model (IAM) and the Stepwise Mutation Model (SMM), and mutation rates (*μ*) for each locus were estimated from the data.

## Results

Relatively high levels of genetic variation were found in *V. reitzii* populations (Table 2). The number of alleles at each locus ranged from 28 to 43, and the allelic richness ranged from 3.589 to 5.531, both in SMPR and CASC, respectively. The observed heterozygosity ranged from 0.411 to 0.499, with the expected heterozygosity ranging from 0.452 to 0.629. With the exception of PDSC, all populations had private alleles. Inbreeding coefficients ranged from 0.089 to 0.310, and with the exception of SMPR, all populations departed significantly from HWE with an excess of homozygotes (Table 2). Micro-Checker analysis detected the presence of null alleles at six loci in different populations (data not shown). No signs of reduction in population size were detected by any of the methods used (Bottleneck analysis and *M*-ratio) for any of the populations investigated.

**Table 2:** Characterization of genetic variability in six populations of *Vriesea reitzii*.

Population	N	*M*-ratio^1^	A	A_P_	*R*_S_	*H*_O_	*H*_E_	*F*_IS_
SMPR	31	0.732	28	1	3.589	0.411	0.452	0.089
CASC	31	0.732	42	4	5.531	0.438	0.629	0.310*
PDSC	30	0.729	31	0	3.846	0.360	0.504	0.286*
LGSC	32	0.733	43	6	5.307	0.499	0.533	0.019*
CSRS	32	0.733	38	1	4.955	0.493	0.571	0.137*
SFRS	31	0.732	40	3	5.100	0.419	0.515	0.167*

N, number of individuals; *M*-ratio, mean ratio of the number of alleles to the range in allele size; A, number of alleles; A_P_, number of private alleles; *R*_S_, allelic richness; *H*_O_, observed heterozygosity; *H*_E_, expected heterozygosity; *F*_IS_, inbreeding coefficient.

1 population is considered to have undergone a bottleneck if its *M*-ratio value falls below the threshold of critical *M*-ratio calculated (0.61). No bottlenecks were detected in the six populations.

* Inbreeding coefficient (*F*_IS_) which departed significantly from Hardy–Weinberg equilibrium (HWE) at the *P* < 0.001 level.

Moderate levels of genetic differentiation were detected among *V. reitzii* populations (*F*_ST_ = 0.123, G’_ST_ = 0.120 and *R*_ST_ = 0.096). The pairwise *F*_ST_ values also suggest low to moderate structure between pairs of populations, and geographical proximity does not seem to be the main factor determining the *F*_ST_ (e.g., CASC and PDSC showed a significant *F*_ST_ = 0.100 (*P* < 0.001) and are 108 km apart, whereas SMPR and CSRS showed a non-significant *F*_ST_ = 0.030 and are 360 km apart (see Figure 1 and Table 3). Accordingly, the Mantel test revealed that geographical distances were not significantly correlated with genetic differentiation as estimated by *r* ^2^ = 0.0001 (*P* = 0.364), suggesting the absence of isolation-by-distance.

**Table 3:** Pairwise genetic divergence (*F*_ST_) below the diagonal and number of effective migrants (*N*_e_m) per generation above the diagonal, for *Vriesea reitzii* populations based on seven microsatellite loci.

	SMRS	CASC	PDSC	LGSC	CSRS	SFRS
SMRS		0.377	0.145	1.161	0.748	0.933
CASC	**0.097**		1.605	1.103	2.541	2.671
PDSC	0.044	**0.100**		0.663	0.584	0.741
LGSC	**0.129**	0.015	**0.150**		1.611	1.200
CSRS	0.030	0.029	0.039	**0.048**		0.923
SFRS	**0.066**	**0.129**	**0.106**	**0.169**	0.043	

*F*_ST_ values in bold were significant at *P* < 0.001.

AMOVA results indicated that the largest percentage of variation (87.42%) was attributed to the within populations component and only a small portion of the genetic variance (12.58%) was attributed to the between populations component (*P* < 0.0001).

**Figure 2:**
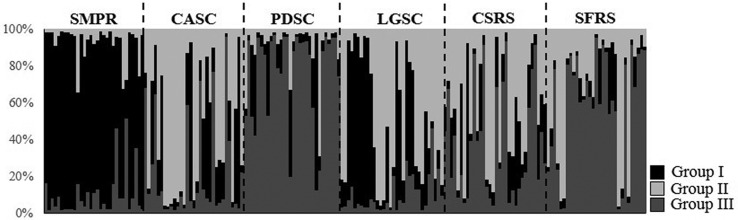
Population structure in *Vriesea reitzii* using Bayesian assignment analysis for a *K* = 3 population model based of seven nuclear microsatellite loci. See Table 1 for population identification.

Bayesian analysis confirmed that a model of *K* = 3 groups best captured the variation in the data from *V. reitzii* (Figure S1). The admixture proportions (*Q*) for each individual are shown in Figure 2. The number of migrants per generation in *V. reitzii* populations varied from 0.145 to 2.671 (Table 3), with approximately half of the comparisons between populations pairs exceeding 1, which is equivalent to > 1 migrant per generation. The minimum migration required for maintaining species cohesion has traditionally been regarded to be one migrant per generation (Morjan and Rieseberg, 2004). Confidence intervals for the effective number of migrants (*N*_e_m) are presented in Table S2.

## Discussion

### Genetic diversity within populations

We investigated the genetic diversity and population structure of *V. reitzii*, a species endemic to the MOF, by using nuclear microsatellite markers. Mountains, islands and even forest fragments are often rich in endemism, but these habitats are commonly at risk of depauperization, causing a reduction of genetic diversity of its populations (Kruckeberg and Rabinowitz, 1985). According to Furtado and Menini-Neto (2015), the particular features of mountainous regions confer them high indices of species richness and cause them to harbor important forest remnants in the form of vegetation islands. However, these ecosystems can be very sensitive to anthropogenic disturbances (Zhao and Gong, 2015). Here, despite the fact that *V. reitzii* is endemic to the southern region of Brazil and restricted to the MOF, a highly particular mountain environment with high anthropogenic influence, we did not find low levels of genetic diversity in the populations sampled across its geographical range. Our results revealed that the levels of genetic diversity in this species are relatively high (Table 2). Although a direct comparison of genetic diversity levels between species is complicated by the use of different methods, the levels of diversity found for *V. reitzii* can be considered high when compared to other studies on Bromeliaceae that are based on many of the same SSR loci (Table S1) used in the present study (Palma-Silva *et al.*, 2009; Zanella *et al.*, 2011, 2016; Cascante-Marín *et al.*, 2014; Lavor *et al.*, 2014; Goetze *et al.*, 2015, 2016). The genetic diversity encountered within *V. reitzii* populations suggests that these populations have not yet been impacted by habitat fragmentation, and genetic drift has not yet decreased within-population genetic diversity. Therefore, although the MOF has suffered a long history of natural fragmentation (Fonseca *et al.*, 2009), *V. reitzii* populations have been able to maintain a moderate to high genetic diversity.

Many recent studies have demonstrated that the rarity or endemicity of a species is not necessarily synonymous with low genetic diversity. Instead, genetic diversity can be influenced by species life-history traits, such as a recent origin from widespread congeners, hybridization, maintenance of genetic diversity within refugial populations, as well as ecological traits, habit and mating system (Torres-Díaz *et al.*, 2007; Ægisdóttir *et al.*, 2009; Hardcastle and Gentry, 2009; Eliades *et al.*, 2011; Goetze *et al.*, 2015; Turchetto *et al.*, 2016). The relatively high genetic diversity found in populations of *V. reitzii* (*R*_S_ = 3.589-5.531 and *H*_O_ = 0.360-0.499) may be related to its life history, such as clonality and an outcrossing reproductive system. Outcrossing plants generally have high within-population genetic diversity (Hamrick and Godt, 1996), and clonal propagation can increase the longevity of genets (Orive, 1993; Goetze *et al.*, 2015). The combination of genet longevity and outcrossing may maintain genetic diversity in fragmented populations (Xiao *et al.*, 2015). The reproductive biology of *V. reitzii* and its breeding system have not yet been studied. However, we assume that it has a mixed mating system, like most *Vriesea* species studied to date (Zanella *et al.*, 2012; Lavor *et al.*, 2014). We also presume that, like its sister species *V. phillipocoburgii* and *V. altodaserrae*, it is pollinated by hummingbirds (Machado and Semir, 2006). Moreover, although the species’ clonality has not yet been formally studied, we observed evident clonal reproduction on our field trips.

Another factor potentially explaining the occurrence of high levels of genetic diversity in this mountain species is that its population may have been founded by multiple genetically diverse individuals. This initial diversity may have been maintained in the stable mountainous conditions throughout the Quaternary glacial/interglacial cycles (Behling and Pillar, 2007), thanks to a combination of relatively large population sizes and recurrent interpopulation gene flow, as reported in the terrestrial orchid *Oreorchis patens* from the Korean mountains (Chung *et al.*, 2012). Since *V. reitzii* is highly associated with the *Araucaria* forest, the expansion of the *Araucaria* forest over the Southern Brazilian Highlands from the late Quaternary until ~1500–1000 years ago (Behling *et al.*, 2004) might have increased population connectivity, thereby representing one possible reason for the great allelic richness encountered here.

Despite their high levels of genetic diversity, almost all populations significantly departed from HWE. Heterozygote deficiency may result from many factors, such as genetic drift and inbreeding. Alternatively, it can occur due to the presence of null alleles (Lavor *et al.*, 2014; Turchetto *et al.*, 2016). We found null alleles at all loci in different populations; however, to relate the excess of homozygotes to biparental inbreeding or drift, we would need to have access to demographic data of the populations. Assuming that *V. reitzii* has a mixed mating system, as many other species of the genus *Vriesea* (Paggi *et al.*, 2007; Lavor *et al.*, 2014), mating among relatives may potentially occur within populations. In the only existing record on ecological and demographic aspects of *V. reitzii*, Favretto and Geuster (2012) report that the species is rare in environments with very dense vegetation, but abundant in places with more dispersed trees, and that it is one of the few species that colonizes *Araucaria angustifolia* in the region of Joaçaba, Santa Catarina, Brazil. In the same study, the authors describe the occurrence of 360 individuals across 2000 m^2^, demonstrating that, wherever they occur, *V. reitzii* populations are abundant; this is consistent with our own observations. High population densities may influence the amount of pollen available for outcrossing in mixed mating species and may have an affect on the rate of selfing and inbreeding (Duminil *et al.*, 2016). In the congener *V. minarum,* an excess of homozygotes has been attributed to selfing or biparental inbreeding, since that species has a mixed reproductive strategy (Lavor *et al.*, 2014). Considering that similar results had been found in many species studied so far, Lavor and colleagues also speculated that their findings might reflect a general pattern characteristic of the family.

It would be interesting to compare the patterns of diversity found in the present study with those of other species endemic to the MOF, however, our exhaustive search turned up very few studies with a genetic focus, and most of these were on *Araucaria angustifolia* (Auler *et al.*, 2002; Bittencourt and Sebbenn, 2007; Medina-Macedo *et. al*., 2015). *Dicksonia sellowiana* is a species of the MOF whose populations were severely reduced due to direct exploitation. Montagna *et al.* (2012) compared the genetic diversity of populations from conservation units to that of populations outside conservation units and found a significant difference in *Ho* between these two groups. Martins *et al.* (2015) studied populations of *Ocotea* spp. in protected and unprotected areas of the MOF and found high *H*o indexes and a small heterozygous deficit. However, none of the studies focused on understanding the dynamics and genetic structure of populations from the mosaic landscape characterizing the MOF, highlighting the importance of the present work in contributing to an improved understanding of the dynamics of MOF species.

### Genetic differentiation among populations

Mountainous and fragmented environments are characterized by landscape heterogeneity, with the mountain ridges or mosaic formations expected to represent major barriers to gene flow among populations (Zhan *et al.*, 2009; Frei *et al.*, 2010). However, against this expectation, we observed an average *F*_ST_ of 0.123 and an average *R*_ST_ of 0.0964, indicating only a moderate differentiation among *V. reitzii* populations (Balloux and Lugon-Moulin, 2002). The similarity between *F*_ST_ and *R*_ST_ estimates suggests that drift and mutation are jointly responsible for the observed level of population differentiation (Balloux and Lugon-Moulin, 2002). The results of studies on differentiation among populations in this kind of landscape can vary widely from species to species (Byars *et al.*, 2009). In the Bromeliaceae, various studies have estimated the variation among populations from outcrops and inselbergs. Domingues *et al.* (2011), Barbará *et al.* (2009) and Boisselier-Dubayle *et al.* (2010) found a high level of population structure in several bromeliad species. Other authors have reported moderate indices of genetic structure, similar to our results for *V. reitzii* (Ribeiro *et al.*, 2013; Lavor *et al.*, 2014).

No correlation between genetic and geographic distances was detected by the Mantel test (*r* ^2^ = 0.0001, *P* = 0.364), indicating an absence of isolation-by-distance among *V. reitzii* populations. Furthermore, Bayesian analysis revealed that *V. reitzii* comprises three genetic groups (Figure 2 and Figure S1). These did not correlate to the geography of sampling localities, and we could find individuals with a predominance of the three genetic components in almost all populations (Figure 2). An evaluation of the region’s topography did not reveal any obvious features that could have led to the formation of the groups found. The next step will be to use plastidial markers in a phylogeographic approach to assess the historical structure of the populations and indicate possible vicariance events underlying the observed pattern. The weak genetic structure between groups, paired with the high number of migrants per generation found in at least half of the population pairs (Table 3), points towards reasonable levels of gene flow among localities. In order to explain this moderate structure and the estimated effective number of migrants, it is possible to relate them to ancient and contemporary factors.

Concerning contemporary gene flow, to date, there are no studies on the pollinator and seed dispersal mechanisms of *V. reitzii*. However, if the species is pollinated by hummingbirds, an effective contemporary gene flow among the populations sampled may occur. Hummingbird behavior varies between species, but some species can migrate up to 2000 km, enough to cover the inter-population distances of *V. reitzii*, which range from 38 km to 360 km (Berthold *et al.*, 2013). Furthermore, *V. reitzii* has plumose seeds (LES personal observation; Leme and Costa, 1991), which is characteristic of the subfamily Tillandsioideae (Benzing, 1990) and can facilitate seed dispersal by wind over long distances. In a study of the bromeliad *V. gigantea*, an epiphyte inhabiting the understory of the Atlantic Forest, Paggi *et al.* (2010) showed that seed dispersal occurs over short distances, although the species’ seeds are plumose and wind-dispersed, as they probably are in *V. reitzii*. On the other hand, Lavor *et al.* (2014) reported that seed dispersal in *V. minarum* is more effective and not directly comparable to the results of Paggi *et al.* (2010). *Vriesea minarum* is restricted to rocky systems in open environments, where the high incidence of winds can enhance seed dispersal. In addition, the usually low stature of the rupicolous vegetation poses fewer obstacles to dispersal than a forested area.

With regard to ancient patterns of gene flow, the current design of genetic structure may have been modeled by past events, such as the expansion of the *Araucaria* forest during the Holocene (Behling and Pillar, 2007) that may have contributed to decrease the distance between populations and increase the gene flow between them. Chung *et al.* (2012), studying orchids of the genus *Oreorchis*, suggested a scenario of high levels of historical gene flow among neighboring populations along the main mountainous ranges in the Korean Peninsula. In a study on the altitudinal gradients of *Silene ciliate*, García-Fernández *et al.* (2012) proposed that the high genetic similarity among populations from different mountains may be due to historical population movements related to glacial contractions and expansions throughout the Quaternary period. Many montane-adapted species exist as isolated populations under todays relatively warm climate; during cooler glacial cycles, these species would have experienced range expansions, increasing population connectivity and gene flow (Devitt *et al.*, 2013). Moreover, both the results of the heterozygosity excess test (Bottleneck program) and the *M*-ratio found here indicate that *V. reitzii* populations did not suffer a bottleneck or reduction of genetic diversity (Table 2), emphasizing that factors involved in the evolutionary history of the MOF might have significantly contributed to genetically homogenize the populations. In this context, it would be interesting to collect data from additional species from the MOF in order to further investigate this hypothesis.

Therefore, the current genetic pattern of *V. reitzii* populations may have been shaped by a range of factors, including the mosaic landscape of alternating grasslands and forests. The *Araucaria* Plateau, where we sampled *V. reitzii* populations, is formed by lightly to markedly undulating terrain at altitudes between 600 and < 1400 m. The MOF’s current mosaic landscape has been shaped by the phases of forest expansion and retraction resulting from climatic changes during the Holocene (Calegari *et al.*, 2017). Coupled with the intense fragmentation of this environment in the twentieth century (Fonseca *et al.*, 2009), the patchwork pattern of this landscape may have influenced the current patterns of diversity and population structure found in *V. reitzii*. In summary, the moderate structure encountered in *V. reitzii* may be due to ancient gene flow, facilitated dispersal mediated by the mosaic landscape of the MOF, or a combination of these two factors.

### Implications for Conservation

*Vriesea reitzii* populations showed high genetic diversity and moderate genetic structure, indicating no imminent threat to this bromeliad. However, as an epiphyte, this species is highly associated with the MOF, and the conservation of this landscape is therefore paramount for the species’ maintenance. Only 12.6% of the *Araucaria* Forest’s original cover remains, and those remnants are mostly distributed in small fragments amid various anthropogenic habitats, such as pasture, agriculture and exotic tree monocultures of *Pinus* and *Eucalyptus* (Ribeiro *et al.*, 2009; Emer and Fonseca, 2011). The *Araucaria* Forest should therefore be considered as critically threatened.

Despite the moderate levels of genetic differentiation between *V. reitzii* populations, almost all have private alleles (Table 2), emphasizing the importance of the conservation of this genetic diversity. The CASC and LGSC populations have four and six private alleles respectively; they therefore deserve special attention in any conservation measures.

If the mixed mating system in *V. reitzii* is confirmed, with at least partial dependence on pollinators, strategies for the maintenance of gene flow among forest fragments should be put in place. Studies on reproductive biology and pollination will be essential to paint a complete picture of the biology of this species.

## Supplementary material

The following online material is available for this article:

Figure S1 - Magnitude of Δ*K* from structure analysis of *K*.

Table S1 - Genetic characterization for seven microsatellite loci.

Table S2 - Confidence intervals of number of effective migrants (*N*_e_m) estimated for *Vriesea reitzii* populations based on seven microsatellite loci.
