# Supplementary material for: Genetic diversity and population structure of Vriesea reitzii (Bromeliaceae), a species from the Southern Brazilian Highlands
Source: Genet Mol Biol. 2018 Mar 19;41(1 Suppl 1):308–17. doi: 10.1590/1678-4685-GMB-2017-0062 (PMC5913716; doi:10.1590/1678-4685-GMB-2017-0062)
Supplement: Supplementary file 1 [file 1415-4757-GMB-41-01-2017-0062-s001.pdf]

Supplementary Material to "Genetic diversity and population structure of *Vriesea reitzii* (Bromeliaceae), a species from the Southern Brazilian Highlands"

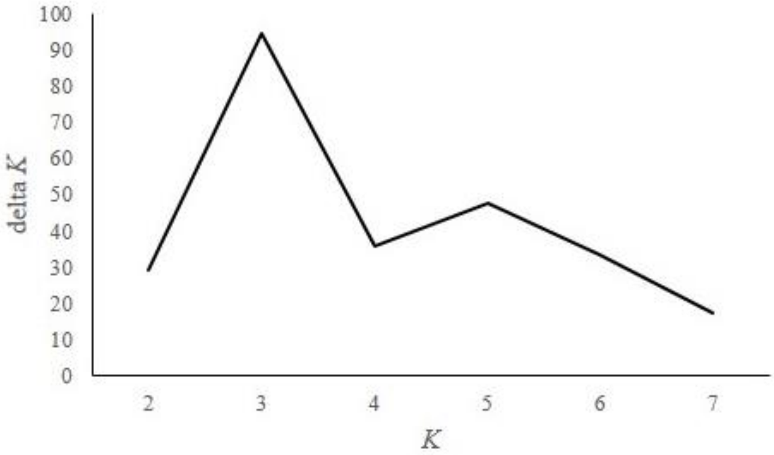

Figure S1 - Magnitude of Δ*K* from structure analysis of *K* (mean ± SD over 10 replicates), calculated by following the Δ*K* method proposed by Evanno *et al.* (2005), for *Vriesea reitzii* microsatellite data. The modal values of these distributions indicate the true *K* or the uppermost level of structure is three "genetic clusters".
