# Supplementary material for: Genetic diversity and population structure of Vriesea reitzii (Bromeliaceae), a species from the Southern Brazilian Highlands
Source: Genet Mol Biol. 2018 Mar 19;41(1 Suppl 1):308–17. doi: 10.1590/1678-4685-GMB-2017-0062 (PMC5913716; doi:10.1590/1678-4685-GMB-2017-0062)
Supplement: Supplementary file 2 [file 1415-4757-GMB-41-01-2017-0062-s003.pdf]

Supplementary Material to "Genetic diversity and population structure of *Vriesea reitzii* (Bromeliaceae), a species from the Southern Brazilian Highlands"

Table S2 - Confidence intervals of number of effective migrants (N_em) estimated for *Vriesea reitzii* populations based on seven microsatellite loci.

Populations	Lower interval (0.050 percentile)	Higher interval (0.095 percentile)
SMPS x CASC	0.275	0.672
SMPS x PDSC	0.103	0.198
SMPS x LGSC	0.919	1.481
SMPS x CSRS	0.569	1.008
SMPS x SFRS	0.709	1.230
CASC x PDSC	1.286	1.992
CASC x LGSC	0.875	1.387
CASC x CSRS	2.059	3.169
CASC x SFRS	2.134	3.346
PDSC x LGSC	0.457	0.870
PDSC x CSRS	0.450	0.756
PDSC x SFRS	0.589	0.924
LGSC x CSRS	1.275	2.014
LGSC x SFRS	0.923	1.531
CSRS x SFRS	0.689	1.175
