# Supplementary material for: Genetic diversity and population structure of Vriesea reitzii (Bromeliaceae), a species from the Southern Brazilian Highlands
Source: Genet Mol Biol. 2018 Mar 19;41(1 Suppl 1):308–17. doi: 10.1590/1678-4685-GMB-2017-0062 (PMC5913716; doi:10.1590/1678-4685-GMB-2017-0062)
Supplement: Supplementary file 3 [file 1415-4757-GMB-41-01-2017-0062-s002.pdf]

Supplementary Material to "Genetic diversity and population structure of *Vriesea reitzii* (Bromeliaceae), a species from the Southern Brazilian Highlands"

Table S1 - Genetic characterization for seven microsatellite loci in six populations of *Vriesea reitzii*.

Locus	Repeat Motif	Allele range size (bp)	A	R_S	H_O	H_E	F_{IS}
e6b	$(CAA)_{12}$	143 - 177	11	6.192	0.334	0.421	0.202*
e19	$(CT)_{15}$	137 – 143	3	2.088	0.340	0.347	0.017
PaA10	$(ATG)_{10}$	169 – 187	3	2,166	0.344	0.391	0.121
VgB10	$(AG)_{24}$	158 – 186	13	10.159	0.598	0.769	0.235*
VgC01	$(CT)_{16}$	218 – 276	10	4.878	0.529	0.596	0.110*
VgF02	$(CT)_{12}(CT)_{17}$	189 – 227	20	12.415	0.651	0.844	0.227*
VgF05	$(CA)_9(A)_{11}$	237 - 243	3	2.288	0.259	0.370	0.299*

* Inbreeding coefficient (F_{IS}) which departed significantly from Hardy–Weinberg equilibrium (HWE) at the $P < 0.001$ level.
